# The effects of cadmium and copper on embryonic and larval development of ide *Leuciscus idus* L.

**DOI:** 10.1007/s10695-013-9832-4

**Published:** 2013-07-25

**Authors:** Malgorzata Witeska, Piotr Sarnowski, Katarzyna Ługowska, Ewelina Kowal

**Affiliations:** Department of Animal Physiology, University of Natural Sciences and Humanities, Prusa 12, 08-110 Siedlce, Poland

**Keywords:** Fish, Embryos, Larvae, Heavy metals, Toxicity

## Abstract

The effects of Cd and Cu on embryos and larvae of the ide *Leuciscus idus* were evaluated. The ide is an European cyprinid fish, natural populations of which tend to decrease. The ide is also used as a bioindicator organism to evaluate water quality. However, sensitivity of ide early developmental stages to heavy metal intoxication is not known. Fish were exposed to Cd or Cu (100 μg/L) during embryonic, larval or both developmental periods. Survival of the embryos, time of hatching, size and quality of newly hatched larvae were evaluated at the end of embryonic period. Correctly developed larvae from the control and Cd or Cu-exposed groups were transferred to clean water, Cd or Cu solutions (100 μg/L) immediately after hatching. Larval development was observed, and the larvae were photographed. Time of yolk sac resorption, onset of active feeding and swim bladder inflation were evaluated, and the measurements were done on body and swim bladder size. The results showed that exposure of embryos to Cd and Cu significantly reduced embryonic survival and increased frequency of body malformations and death in newly hatched larvae and delayed hatching. Exposure to Cd and Cu during larval period reduced larval survival, growth and delayed development (yolk utilization, beginning of active feeding and swim bladder inflation). Cadmium was more toxic to the ide embryos and larvae than copper. Exposures to metals during embryonic period alone caused adverse impact on larval performance even when larval development took place in clean water. However, exposure of embryos to Cu reduced toxic impact of metal on larvae in continuous Cu exposure compared to the non-preexposed fish, but no such an effect occurred in case of Cd exposure. The results show that even a short-term exposure to Cd or Cu during early development of ide may adversely affect recruitment of this species. Among the measured endpoints, quality of newly hatched larvae (frequency of body malformations and larvae dead immediately after hatching) and swim bladder size were the most sensitive to intoxication with both metals. Embryos were more sensitive to Cu intoxication than larvae, while in case of Cd, sensitivity of both stages was similar.

## Introduction

The ide (*Leuciscus idus* L.) is a mostly riverine cyprinid fish species inhabiting large and medium size rivers in Europe and Asia. They are planktivorous and benthivorous, of average body size of 40–50 cm, prefer deep, clean and cool water and reach sexual maturation at the age of 3–4 years. Spawning takes place from April to June at sandy or gravel spawning substrate. According to Hamackova et al. (2007), in some European countries (e.g., Czech Republic), the ide belongs to less frequent and vulnerable species; thus, it is bred in captivity and reared under controlled conditions. Also, in Poland, natural populations of many rheophilic fish including the ide are dwindling due to environment deterioration, and the fish are artificially reproduced and prereared under hatchery conditions (Wolnicki and Górny 1995) and cultured in carp farms (Cieśla and Wojda 2004; Krejszeff et al. 2009). *L. idus* is also used to assess the quality of surface water (Allner et al. 1999; Fenske et al. 2006) and for toxicity tests (Braunbeck and Segner 1992; Lenyen et al. 1998). However, all these studies were performed on juvenile or adult fish, while little is known about sensitivity of the early life stages of ide to environmental contaminants.

Fish embryos and larvae are more sensitive to environmental impacts, including toxic substances compared to the juveniles and adults (Dave and Xiu 1991). Various embryonic morphological, physiological and behavioral endpoints are used to assess toxicity of chemicals (Weis et al. 2003; Fraysse et al. 2006). Fish larvae are also a useful animal model for toxicity evaluation since they are small but developed enough to have almost all functional organs (Hernandez and Allende 2008). Sensitivity of various early developmental stages to intoxication is different, and so is sensitivity of various endpoints (Nguyen and Janssen 2002; Jezierska et al. 2009a, b).

Cadmium is a xenobiotic, while copper is an important essential metal. However, both are widespread and highly toxic environmental pollutants. Copper sulfate is also used in fish culture to control saprolegniosis on eggs (Straus et al. 2012) and as an antibacterial agent for treatment of larvae (Chen et al. 2006). Various studies showed that sublethal concentrations of these metals are toxic to fish embryos and larvae, and various physiological disturbances were observed.

In unpolluted freshwaters, the levels of Cd and Cu range from 0 to 13 and 2 to 4 μg/L, respectively. Contaminated waters often contain much more: 40–120 μg/L of Cd and 1–137 μg/L of Cu (Dojlido 1995). Cadmium and copper under acidic conditions are leached from the sediments, so, the concentrations of free Cu^2+^ and Cd^2+^ ions may temporarily increase. Spring snowmelt often acidifies water; thus, the risk of contamination coincides with spawning and development of early stages of fish. According to Zyśk (2013), sediments of Vistula River contain 2 mg/kg of Cd and 12 mg/kg of Cu, while Adamiec and Helios-Rybicka (2002) reported 3–21 mg/kg of Cd and 31.3–298 mg/kg of Cu in sediments of Odra River.

According to Paquin et al. (2002), acute toxic action of metals involves ionoregulatory disturbances by affecting the activity of Na^+^/K^+^ATPase and displacement of calcium from paracellular junctions which results in increased permeability of epithelia and in consequence in sodium loss from the organism.

There are many data showing toxicity of cadmium to early life stages of various fish species. Short-time pulse-exposure of *Melanotaenia fluviatilis* embryos and larvae to 3,300 μg/L of Cd resulted in embryonic deformities reduced percentage of hatched larvae and larval spinal deformities (Williams and Holdway 2000). According to Ismail and Yusof (2011), exposure of fertilized eggs of *Oryzias javanicus* to low levels of cadmium (10–50 μ/L) caused developmental disorders, while concentration of 100 μ/L completely inhibited development and resulted in death of all embryos. Lizardo-Daudt and Kennedy (2008) reported disturbances of hatching, reduced growth and endocrine disruption in *Oncorhynchus mykiss* larvae subjected from fertilization to low concentrations of cadmium (0.05–2.5 μg/L). Developmental defects and mortality were observed in embryos and pro-larvae of *Silurus soldatovi* exposed to various concentrations of cadmium (Zhang et al. 2012). Cadmium was also observed to induce oxidative stress in *Paralichthys olivaceus* larvae (Cao et al. 2010) and directly affected membrane calcium transport in *Danio rerio* larvae which resulted in reduced Ca^2+^ uptake (Liu et al. 2012).

Toxicity of copper to early developmental stages of fish is also well documented. Chen et al. (2012) reported that even short-term pulsed exposure to low levels of copper reduced growth rate of *Oreochromis mossambicus* larvae. Nguyen and Janssen (2002) reported that copper reduced growth and caused morphological anomalies in *Clarias gariepinus* larvae. According to Johnson et al. (2007), copper exposure (11–1,000 μg/L) of *D. rerio* embryos resulted in mortality, hatching inhibition, impairment of larval development and lateral line dysfunction. The latter effect of copper was also reported by (Hernandez et al. 2006 and Linbo et al. 2006, 2009).

Barjhoux et al. (2012) reported that both cadmium and copper induced morphological anomalies and genotoxic effect in *Oryzias latipes* embryos and larvae. Sikorska and Wolnicki (2010) exposed *Tinca tinca* larvae to 100–300 μg/L of cadmium or copper and observed reduced growth and survival and retarded swim bladder inflation. The highest concentrations of both metals delayed the onset of exogenous feeding. According to Alsop and Wood (2011), there is a common pathway for uptake of several heavy metals including cadmium and copper and a common mechanism of toxicity, probably total ion loss by diffusion.

Some data suggest the possibility of acclimation of early stages of fish to cadmium and copper by pretreatment due to stimulation of metallothionein synthesis (Wu and Hwang 2003).

Our previous studies showed that cadmium and copper at the concentrations of 100 μg/L adversely affected embryonic and larval survival and development of various species of cyprinid fish: *Cyprinus carpio* and *Barbus barbus* (Jezierska and Slominska 1997; Slominska and Jezierska 2000; Jezierska et al. 2009a, b; Witeska et al. 2010; Ługowska and Kubik 2011).

The aim of present study was to evaluate the effects of cadmium and copper on embryonic and larval development of the ide *L. idus* and to compare sensitivity of various developmental stages and endpoints to toxic action of these metals.

## Materials and methods

The eggs and sperms of the ide were obtained from the hatchery of Samoklęski Fish Farm in Kamionka, Poland. Chilled gametes were transported to the laboratory in the cold box at 5 °C for about 2 h. Then the eggs and sperms were gradually warmed up to the ambient temperature and in vitro fertilization took place. Fertilized eggs were placed in glass Petri dishes and these—in 2 L aquaria. Water was constantly gently aerated, and constant temperature 16 °C was maintained using thermostat, pH was 7.8–8.0, total hardness 178 mg/dm^3^ as CaCO_3_, DO saturation ≥80 %, NH_4_^+^ 50 μg/L, NO_2_^−^ 5 μg/L. Clean non-chlorinated tap water used in the experiment contained <1 μg/L of Cd and <40 μg/L of Cu and 4.0 mg/L of Na and 55.7 mg/L of Ca. Three experimental groups were created: control—the embryos were incubated in clean water, Cd—incubation took place in water containing 100 μg/dm^3^ of cadmium (as CdCl_2_ × 21/2H_2_O) and Cu—in water containing 100 μg/dm^3^ of copper (as CuSO_4_ × 5H_2_O). Each group consisted of 4 dishes (replicates), on average 161 (146–183) eggs in each. Water was changed every day by gentle siphoning out and replacement with fresh clean water (control) or fresh metal solutions (Cd and Cu). During embryonic period development, observations were made twice a day and dead embryos were counted and removed (opaque eggs). Time of hatching and final survival of newly hatched larvae was calculated. The quality of newly hatched larvae was also evaluated, and percentage was calculated of correctly developed and viable, deformed (showing various morphological anomalies such as vertebral curvatures and yolk sac malformations), and dead—those that died immediately after hatching.

Correctly developed and viable newly hatched larvae were transferred to 14 L aerated aquaria and divided into 7 experimental groups, 50 larvae in each (Table 1). Every day, the larvae were gently harvested with the plastic sieve, counted and transferred to clean aquarium with fresh water or metal solution, respectively. At the same time, dead larvae were also counted and removed. During larval development, constant water temperature 18 °C was maintained, pH was 7.8–8.0, water hardness 178 mg/dm^3^ as CaCO_3_ and DO saturation ≥80 %). Constant gentle aeration was applied. Beginning from the 3 day post-hatching (dph), the larvae were fed 3 times a day *Artemia* nauplii ad libitum. Experimental rearing lasted 21 days.

**Table 1:** Experimental groups and their development conditions

Group	Embryonic development	Larval development	Number of fish
Control	Clean water	Clean water	50
Cd–Cd	Cd 100 μg/L	Cd 100 μg/L	50
Cu–Cu	Cu 100 μg/L	Cu 100 μg/L	50
Cd–0	Cd 100 μg/L	Clean water	50
Cu–0	Cu 100 μg/L	Clean water	50
0–Cd	Clean water	Cd 100 μg/L	50
0–Cu	Clean water	Cu 100 μg/L	50

Immediately after hatching, 30 larvae from each group (Control, Cd and Cu) were photographed using stereoscopic microscope connected with the camera and computer with MultiScan image analysis system (Computer Scanning Systems, Poland). During larval exposure, 20 larvae from each group (Control, Cd–Cd, Cu–Cu, Cd–0, Cu–0, 0–Cd and 0–Cu) were photographed daily until the end of the experiment (21 dph). The larvae were individually placed in a watch glass with water or metal solution under microscope; magnification was adjusted to the increasing size of larvae, and the scaled photographs were taken; then the larvae were returned back to the aquaria. Entire procedure for each larva took about 1 min. All measurements were done in the photographs: body length, body perimeter area (excluding yolk sac), yolk sac perimeter area and swim bladder perimeter area (separately for posterior and anterior chamber). For evaluation of the effects of Cd and Cu on the shape of larvae, the ratio of body perimeter area to length was calculated for newly hatched and 21-day-old-larvae. For the newly hatched larvae, also, the ratio of yolk sac perimeter area to body perimeter area was calculated to evaluate relative size of nutrient abundance. Perimeter area of entire swim bladder was made by addition of the results to both chambers. Time of complete yolk sac resorption, first feeding (based on the presence of *Artemia* in the digestive tract) and the beginning of swim bladder inflation was also registered (when at least 50 % of larvae showed the feature). For comparison between the sensitivity of various embryonic and larval endpoints to metal toxicity and of the effects of various exposures, percent change of mean values of the studied parameters in relation to the control was calculated.

The obtained results were subjected to statistical analysis using Statistica 9.1 (Stat Soft). Significance of differences among groups was tested using *t* test at *p* ≤ 0.05. For clarity of the results, only initial and final values were shown.

## Results

**Fig. 1:**
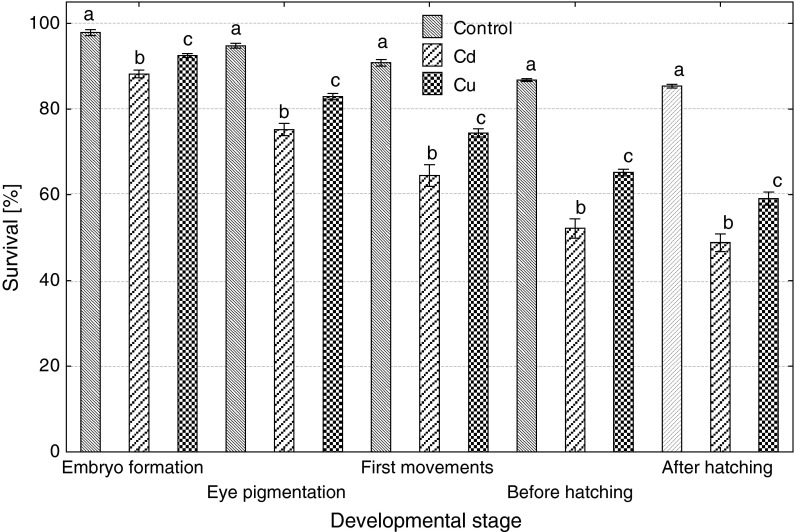
The effects of exposure to 100 μg/L of cadmium or copper on survival of ide embryos at various developmental stages (*different letter* *superscripts* indicate significant differences among experimental groups at each developmental stage, *t* test, *p* ≤ 0.05, *n* = 4)

**Fig. 2:**
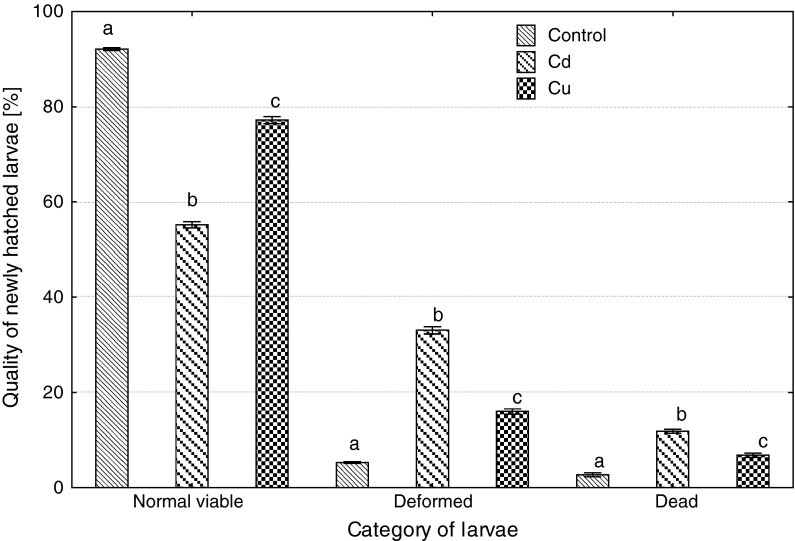
The effects of embryonic exposure to 100 μg/L of cadmium or copper on the quality of newly hatched ide larvae (*different letter* *superscripts* indicate significant differences in percentage of each category of larvae among experimental groups, *t* test, *p* ≤ 0.05, *n* = 4)

**Fig. 3:**
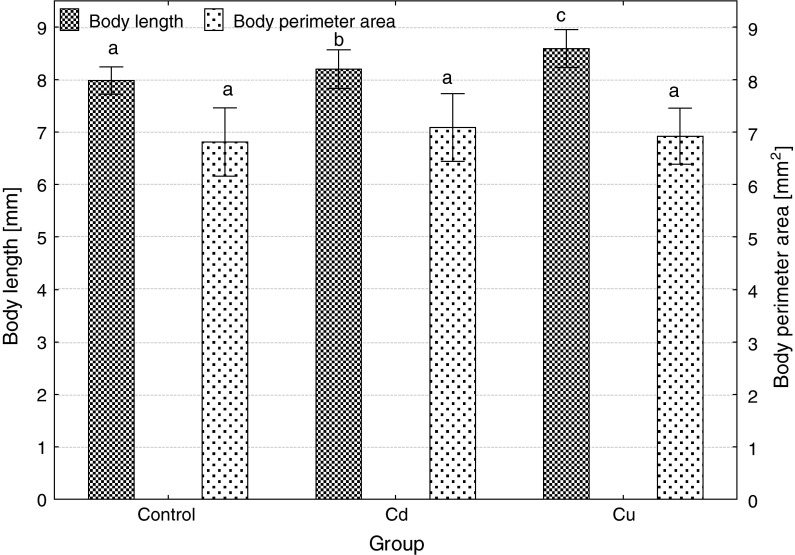
The effects of embryonic exposure to 100 μg/L of cadmium or copper on the size (body length and perimeter area) of newly hatched ide larvae (*different letter* *superscripts* indicate significant differences among experimental groups, *t* test, *p* ≤ 0.05, *n* = 30)

**Fig. 4:**
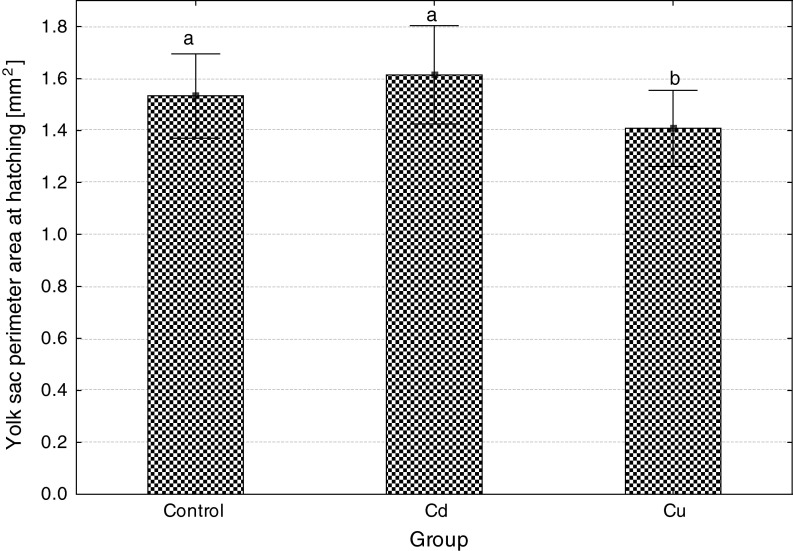
The effects of embryonic exposure to 100 μg/L of cadmium or copper on the size (perimeter area) of the yolk sac in newly hatched ide larvae (*different letter* *superscripts* indicate significant differences among experimental groups, *t* test, *p* ≤ 0.05, *n* = 30)

Embryonic exposure to Cd and Cu significantly reduced survival at each developmental stage, Cd more than Cu (Fig. 1). Both metals caused a delay in the beginning and the end of hatching; in the control, hatching started 114 h post-fertilization (hpf) and ended 135 hpf, while in Cd-exposed group, 125 and 141, and in Cu-exposed group, 128 and 150 hpf. Percentage of deformed and dead larvae significantly increased after Cd and Cu exposure of embryos (Fig. 2), and the effect of cadmium was significantly more pronounced compared to copper. The size of newly hatched larvae also significantly differed (Fig. 3): the shortest larvae hatched in the control, while the longest in Cu-contaminated water, but body perimeter area did not significantly differ among the groups. The ratio of body perimeter area to body length was significantly lower in Cu-exposed group (0.81 ± 0.06) compared to the control (0.85 ± 0.08) and Cd group (0.87 ± 0.10). Newly hatched larvae from Cu group showed significantly smaller yolk sac than fish from the control and Cd group (Fig. 4). The ratio of the yolk sac perimeter area to body perimeter area was 0.23 ± 0.03 in the control, 0.23 ± 0.04 in Cd and only 0.02 ± 0.04 in Cu group (significantly less than in control and Cd).

The lowest larval mortality at the end of the experiment (on 21 dph) occurred in the control group (5 %), while the highest is in the Cd–Cd group (50 %). Mortality in Cd–0 and 0–Cd was 25 and 33 %, respectively, while in Cu-intoxicated groups: Cu–Cu, Cu–0, and 0–Cu: 18, 12, and 27 %, respectively.

**Fig. 5:**
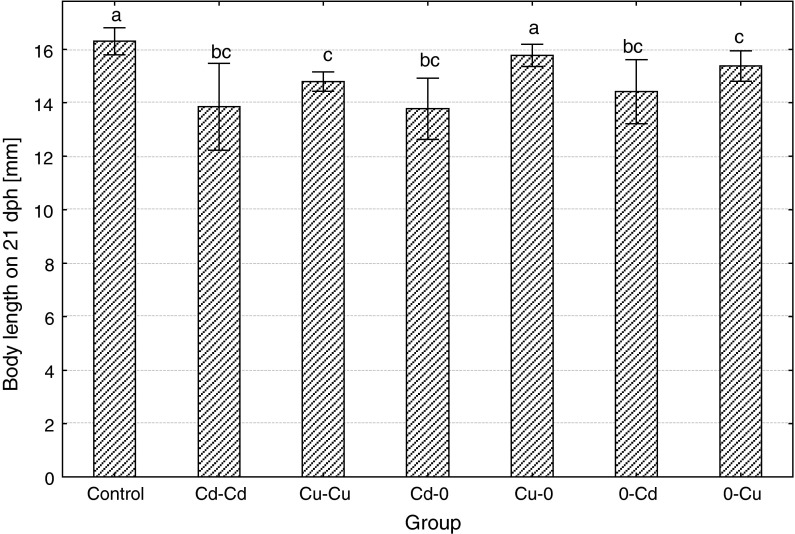
The effects of exposure to 100 μg/L of cadmium or copper during embryonic or larval development or in both periods on body length of the ide larvae on the 21 dph (*different letter* *superscripts* indicate significant differences among experimental groups, *t* test, *p* ≤ 0.05, *n* = 20)

**Fig. 6:**
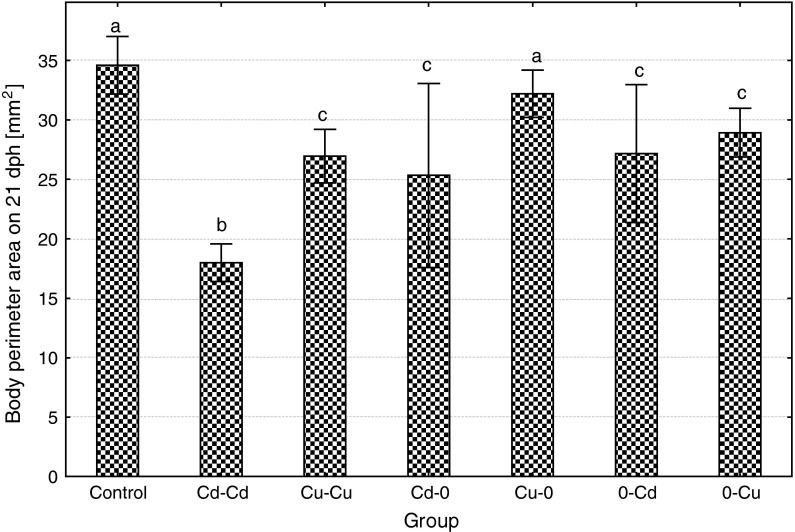
The effects of exposure to 100 μg/L of cadmium or copper during embryonic or larval development or in both periods on body perimeter area of the ide larvae on the 21 dph (*different letter* *superscripts* indicate significant differences among experimental groups, *t* test, *p* ≤ 0.05, *n* = 20)

Body size of larvae at the end of the experiment (21 dph) significantly differed among experimental groups, body length less than perimeter area (Figs. 5, 6). The longest larvae were in the control and similar (slightly but insignificantly shorter) in Cu–0 group, while the fish from Cd–0d were the shortest. Similarly, as the length, also body perimeter area at the end of the experiment was the highest in the control and very similar in Cu–0 group. Fish exposed to cadmium over entire embryonic and larval period (Cd–Cd) were the smallest (with body perimeter area about twice smaller compared to the control), while the ide from Cd–0 and 0–Cd groups were smaller compared to the control but significantly bigger than those from Cd–0d (high individual variability occurred in these groups). The fish from Cu–Cu and 0–Cu groups were smaller than fish from the control but bigger than in Cd–Cd. The ratio of body perimeter area to the length was highest in the control (2.12 ± 0.13). Significantly, lower perimeter to length index was observed in all metal-exposed groups except for Cd–0 (due to high individual variability in this group). The values of the index were: in Cd–Cd 1.32 ± 0.19, in Cu–Cu 1.82 ± 0.16, in Cd–0 1.86 ± 0.61, in Cu–0 2.04 ± 0.10, in 0–Cd 1.87 ± 0.25 and in 0–Cu 1.88 ± 0.11.

All ide larvae from the control and Cu–0 groups completely utilized yolk on 7 dph, fish from Cd–0, 0–Cd and 0–Cu on 9 dph, while those from Cd–Cd and Cu–Cu on 11 dph. First feeding was observed on the 5 dph in the control, on the 7 dph in Cd–0, 0–Cd, Cu–0 and 0–Cu, and on the 9 dph in Cd–Cd and Cu–Cu.

**Fig. 7:**
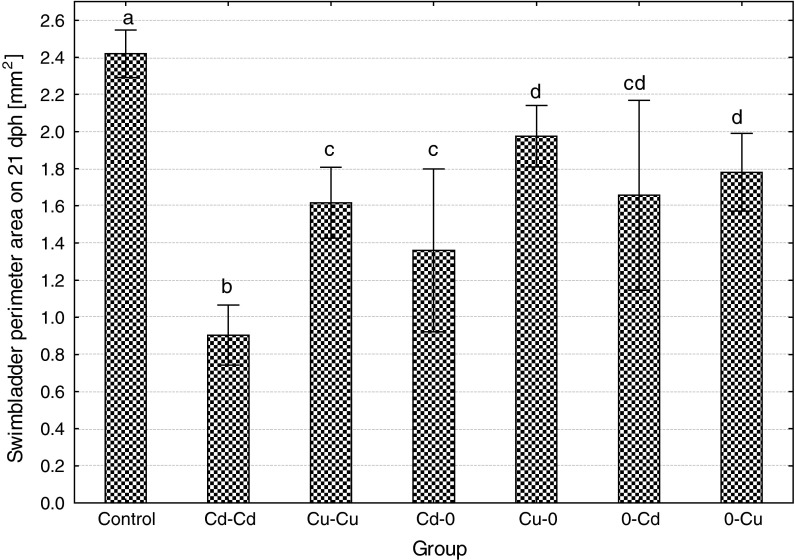
The effects of exposure to 100 μg/L of cadmium or copper during embryonic or larval development or in both periods on the size (perimeter area) of swim bladder in the ide larvae on the 21 dph (*different letter* *superscripts* indicate significant differences among experimental groups, *t* test, *p* ≤ 0.05, *n* = 20)

On the 3 dph, most fish from the control, 0–Cu, Cu–0 and Cd–Cd started to inflate posterior chamber of the swim bladder, while in Cu–Cu, Cd–0 and 0–Cd, the onset of swim bladder inflation took place on the 5 dph. Inflation of the anterior swim bladder chamber started later: on 13 dph in the control and Cu–0, on 15 dph in Cd–0, on 17 dph in and Cd–Cd and Cu–Cu and on 19 dph in 0–Cd and 0–Cu. At the end of the experiment, fish from the control showed swim bladders significantly larger than in all metal-exposed groups, while swim bladder perimeter area of the ide exposed continuously to cadmium (Cd–Cd) was the smallest (only about 30 % of the size in the control) (Fig. 7). The size of posterior and anterior swim bladder chamber showed similar pattern (the results are not shown).

**Fig. 8:**
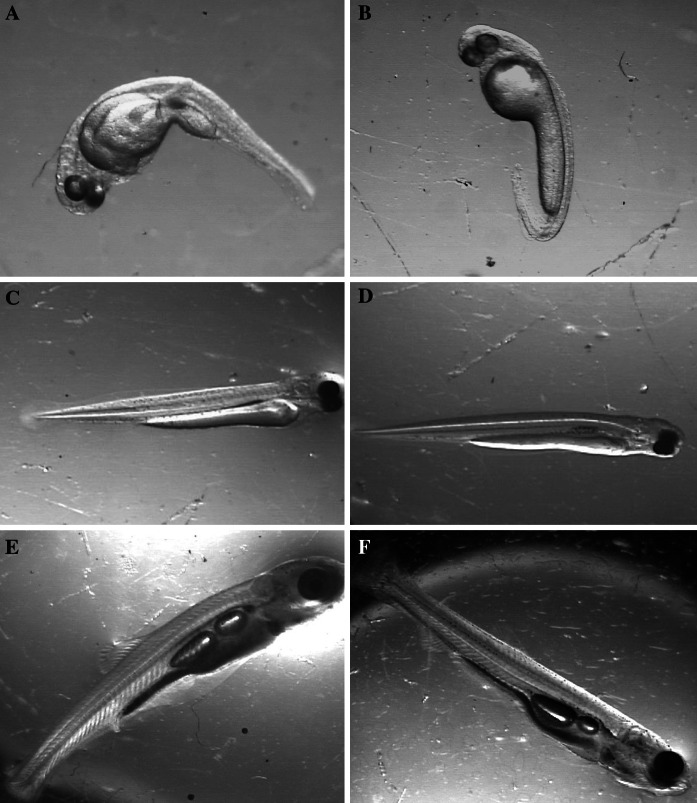
Examples of the most typical effects of Cd and Cu exposures of ide larvae. **a**–**d** Newly hatched, **e**, **f** 21 days old. **a** Deformed from Cd, **b** deformed from Cu, **c** normal from the control (large yolk sac), **d** normal from Cu (small yolk sac), **e** from the control (large swim bladder), **f** from Cd—small swim bladder

The most typical effects of Cd and Cu exposures on ide larvae are shown in Fig. 8.

## Discussion

The obtained results show that copper and cadmium significantly reduced embryonic survival and quality of newly hatched larvae of the ide, cadmium being significantly more toxic compared to copper (Table 2). The results of many studies showed higher toxicity of copper compared to cadmium (literature review: Jezierska and Witeska 2001; Zhu et al. 2011). However, some data indicate that Cd is more toxic to early stages of some fish species than Cu, e.g., Barjhoux et al. (2012) reported that LOEC of Cd and Cu for *O. latipes* embryos were 1.9, and 8.5 μg/L, respectively. Our results obtained for other cyprinid fishes showed that Cu was more toxic than Cd for *C. carpio* embryos and larvae (Jezierska et al. 2009a, b) but Cd was more toxic than Cu for *B. barbus* early life stages (Witeska et al. 2010; Ługowska and Kubik 2011). Similar effect was reported by Sikorska and Wolnicki (2010) for *T. tinca* larvae.

**Table 2:** Sensitivity of various embryonic and larval endpoints to cadmium and copper toxicity (as % of change compared to the value in the control, statistically significant changes—bold, arrows indicate the direction of change)

	Cd	Cu
Embryonic parameters		
Survival at hatching	**42**.**7** **↓**	**30**.**8** **↓**
Frequency of deformed larvae	**535** **↑**	**208** **↑**
Frequency of dead larvae	**337**	**152** **↑**
Body length at hatching	**2**.**8** **↑**	**7**.**6** **↑**
Body perimeter area at hatching	4.1 ↑	1.6 ↑
Yolk sac at hatching	5.2 ↑	**7**.**8** **↓**

	Cd–Cd	Cu–Cu	Cd–0	Cu–0	0–Cd	0–Cu
Larval parameters						
Survival on 21 dph	47.4 **↓**	13.7 **↓**	21.1 **↓**	7.4 **↓**	29.5 **↓**	23.2 **↓**
Body length on 21 dph	**15**.**3** **↓**	**9**.**2** **↓**	**15**.**3** **↓**	3.1 ↓	**11**.**7** **↓**	**5**.**5** **↓**
Body perimeter area on 21 dph	**48**.**0** **↓**	**22**.**0** **↓**	**26**.**9** **↓**	6.9 ↓	**21**.**4** **↓**	**16**.**5** **↓**
Swim bladder perimeter area on 21 dph	**62**.**8** **↓**	**33**.**1** **↓**	**43**.**8** **↓**	**18.2** **↓**	**31**.**4** **↓**	**26**.**4** **↓**

According to Alsop and Wood (2011), toxicity of cadmium and copper involves similar ionoregulatory disturbances. However, these authors observed different levels of reduction in calcium and sodium uptake and body level in *D. rerio* exposed to these metals: Cd reduced Ca uptake more than Cu, while Cu reduced more Na uptake and whole body cation level than Cd. According to Verbost et al. (1989), Cd reduces transepithelial Ca^2+^ influx due to inhibition of basolateral Ca^2+^ATPase and blocking of apical Ca^2+^ channels. This may result in hypocalcemia (Pratap and Wendelaar Bonga 2007) and disturbances of calcium concentration gradient between extracellular and intracellular environment which is essential for various vital functions. Copper was reported to inhibit Na^+^/K^+^ATPase in fish (Li et al. 1998; Kulac et al. 2013). According to Pelgrom et al. (1995), copper causes a decrease in Na influx and Na plasma level in fish. According to McGeer et al. (2000), Cu or Cd-exposed larvae of *O. mykiss* showed significantly reduced body Na^+^ and Ca^2+^ concentrations. Therefore, the differences in sensitivity of various species of fish to toxicity of Cd and Cu are probably related to the intrinsic differences in sensitivity of their calcium and sodium homeostatic mechanisms. This issue, however, requires further detailed studies.

Both metals caused a delay of the beginning and the end of hatching of the ide larvae. Extended development duration of metal-exposed embryos resulted in hatching of significantly longer larvae, particularly in Cu-exposed group. These larvae were also significantly thinner compared to those from the control and Cd-exposed group, and showed the smallest yolk sac size at hatching. This indicates that Cu-exposed embryos consumed the largest amount of nutrients compared to the control and Cd group. Significantly lower relative yolk size (proportion of the yolk to larval body size) suggests higher metabolic expenditure of Cu-exposed embryos. Inhibition of *Brachydanio rerio* hatching by copper was reported by Dave and Xiu (1991). According to Lizardo-Daudt and Kennedy (2008), the effect of cadmium on hatching of *O. mykiss* depended on metal concentration: at 0.05 and 0.25 μg/L premature hatching occurred, while at 2.5 μg/L hatching was delayed. Prolonged hatching time and increased mortality of newly hatched *Pimephales promelas* larvae in metal-contaminated lakes was reported by Gauthier et al. (2006). High rate of yolk utilization in Cu-exposed fish might have been related to activation of metabolic mechanisms of metal sequestration, storage and excretion. According to Romeo et al. (2000), copper is more efficient in activating detoxification mechanisms of fish organism than cadmium. According to various authors, e.g., Roesijadi (1992) and Pelgrom et al. (1995), detoxification of heavy metals in fish involves binding to metallothioneins that participate in homeostasis of essential elements (e.g., Cu) and sequestration of xenobiotics (e.g., Cd).

Cadmium caused stronger increase in frequency of body malformation and higher mortality of newly hatched larvae than copper. Body deformities in *M. fluviatilis* larvae pulse-exposed to cadmium were reported by Williams and Holdway (2000). According to Mochida et al. (2009), vertebral deformities in *Fundulus heteroclitus* larvae exposed to copper pyrithione resulted from inhibition of acetylcholinesterase activity. Mechanism of body malformation in early developmental stages of fish due to metal toxicity is not clear. According to Muramoto (1981), cadmium-induced body deformities in fish were related to a decrease in Ca and P content. Developmental abnormalities may also result from genotoxic action of Cd and Cu which was reported by various authors (e.g., Cavas et al. 2005; Rocha et al. 2010; Ozkan et al. 2011).

Similarly to the embryos, also, the larvae of ide were more affected by cadmium compared to copper: Survival, body length and perimeter area as well as swim bladder size were the most reduced in Cd–Cd group compared to the control (Table 2). Decrease in fish growth caused by copper or cadmium intoxication may be a result of various metabolic disturbances. Couture and Kumar (2003) reported a direct inhibition of mitochondrial enzymatic activity and oxidative metabolism in *Perca flavescens* exposed to copper and cadmium. Metabolic costs of detoxification may also reduce growth of fish exposed to Cd and Cu. According to Wu and Hwang (2003), Cu and Cd exposures induced metallothionein synthesis in *O. mossambicus* larvae. These metals also cause ionoregulatory disorders in fish which may cause increased metabolic cost of compensatory osmoregulation and growth reduction due to energetic deficiency. Slower growth of ide larvae exposed to cadmium and copper might have also resulted from delayed first feeding which occurred 2–4 days later than in the control (except for Cu–0 group). It is noteworthy that only in Cu–0 group body size of 21 dph larvae did not significantly differ from the control. Delayed onset of exogenous feeding in *B. barbus* larvae exposed to 100 μg/L of cadmium or copper was observed by Witeska et al. (2010). The data obtained by Hernandez et al. (2006), Linbo et al. (2006) and Johnson et al. (2007) showed that copper induces lateral line dysfunction in fish larvae. On the other hand, cadmium impaired olfactory sense in *D. rerio* larvae (Kusch et al. 2007). Therefore, growth reduction in Cd- and Cu-exposed fish may result from impaired perception and reduced food uptake.

Almost all groups of larvae exposed to cadmium and copper showed later yolk resorption and the onset of active feeding compared to the control, and the effect was the most pronounced in fish subjected to metal intoxication during embryonic and larval periods. The time of beginning of swim bladder inflation was less affected; however, final size of this organ was considerably reduced in almost all metal-exposed groups. Various authors also observed a delay of yolk absorption in fish larvae intoxicated with heavy metals. According Hwang et al. (1995) observed that *O. mossambicus* larvae exposed to 200 μg/L of copper had larger yolk sacs and were shorter compared to the control. According to Wu et al. (2003a, b) *O. mossambicus* larvae exposed to 30–400 μg/L of copper also showed reduced yolk utilization rate. Johnson et al. (2007) reported that the rate of yolk utilization by the larvae of *D. rerio* intoxicated with Cu at the concentration of 50–1,090 μg/L was reduced in a concentration-related way. Sarnowski (2003) observed delayed yolk sac resorption by *C. carpio* larvae exposed to 200 μg/L of copper or cadmium. According to Sikorska and Wolnicki (2010), larvae of *T. tinca* exposed for 24 h to 100–300 μg/L of Cd or Cu showed reduced growth, survival and retarded swim bladder inflation. The same authors (Sikorska and Wolnicki 2006) reported similar effects accompanied by a delayed onset of exogenous feeding in *Scardinius erythrophthalamus* larvae exposed to Cd. According to Stouthart et al. (1996), a delay of yolk utilization in fish larvae intoxicated with heavy metals may result from reduced metabolic rate or from a direct adverse effect of metals on yolk material.

The results show that exposures to copper and cadmium during embryonic period alone caused adverse impact on larval performance, even when larval development took place in clean water. However, survival, growth and development of fish exposed to Cu only during embryonic period (Cu–0) were less affected than in case of embryonic Cd exposure (Cd–0). Also, exposure of embryos to Cu reduced toxic impact of metal on larvae: Mortality on 21 dph in Cu–0u group was lower compared to 0–Cu, but no such an effect was observed for Cd. This indicates the possibility of acclimation to Cu toxicity by preexposure. Little is known about acclimation mechanisms in early developmental stages of fish. Possibility of acclimation of post-swimup larvae of *Salmo trutta* by exposure of embryos or preswimup larvae to cadmium was observed by Brinkman and Hansen (2007). According to Sellin et al. (2005), acclimation of *Pimephales promelas* larvae to Cu required only 4 days of preexposure, while in juveniles, it took 16 days which indicates high acclimation potential at early stages. Wu and Hwang (2003) found that Cd and Cu stimulated metallothionein synthesis in *O. mossambicus* larvae. Induction of metallothionein synthesis in larvae may also result from maternal Cd exposure (Wu et al. 2012).

The results show that cadmium was more toxic to the ide embryos and larvae than copper, and even a short-term exposure to Cd or Cu during early development of ide may adversely affect recruitment of this species. Taking into consideration continuous exposure of ide embryos and larvae to Cd sensitivity of measured endpoints was quality of newly hatched larvae > swim bladder size > survival of 21 dph larvae > survival at hatching > larval body size at 21 dph. In case of continuous Cu exposure, the ranking was quality of newly hatched larvae > swim bladder size > survival at hatching > larval body size at 21 dph > survival of 21 dph larvae.
